# Low Cost Extraction and Isothermal Amplification of DNA for Infectious Diarrhea Diagnosis

**DOI:** 10.1371/journal.pone.0060059

**Published:** 2013-03-28

**Authors:** Shichu Huang, Jaephil Do, Madhumita Mahalanabis, Andy Fan, Lei Zhao, Lisa Jepeal, Satish K. Singh, Catherine M. Klapperich

**Affiliations:** 1 Department of Biomedical Engineering, Boston University, Boston, Massachusetts, United States of America; 2 Department of Biology, Boston University, Boston, Massachusetts, United States of America; 3 Department of Gastroenterology, Boston University School of Medicine, Boston, Massachusetts, United States of America; 4 Department of Medicine, VA Boston Health Care System, Boston, Massachusetts, United States of America; 5 Department of Mechanical Engineering, Boston University, Boston, Massachusetts, United States of America; Robert Koch Institut, Germany

## Abstract

In order to counter the common perception that molecular diagnostics are too complicated to work in low resource settings, we have performed a difficult sample preparation and DNA amplification protocol using instrumentation designed to be operated without wall or battery power. In this work we have combined a nearly electricity-free nucleic acid extraction process with an electricity-free isothermal amplification assay to detect the presence of *Clostridium difficile* (*C. difficile*) DNA in the stool of infected patients. We used helicase-dependent isothermal amplification (HDA) to amplify the DNA in a low-cost, thermoplastic reaction chip heated with a pair of commercially available toe warmers, while using a simple Styrofoam insulator. DNA was extracted from known positive and negative stool samples. The DNA extraction protocol utilized an air pressure driven solid phase extraction device run using a standard bicycle pump. The simple heater setup required no electricity or battery and was capable of maintaining the temperature at 65°C±2°C for 55 min, suitable for repeatable HDA amplification. Experiments were performed to explore the adaptability of the system for use in a range of ambient conditions. When compared to a traditional centrifuge extraction protocol and a laboratory thermocycler, this disposable, no power platform achieved approximately the same lower limit of detection (1.25×10^−2^ pg of *C. difficile* DNA) while requiring much less raw material and a fraction of the lab infrastructure and cost. This proof of concept study could greatly impact the accessibility of molecular assays for applications in global health.

## Introduction

Each year, over 9.5 million deaths are caused by infectious diseases, nearly all occurring in developing nations [1]. Diseases like diarrhea, malaria, and pneumonia are the leading causes of death and disability, particularly in newborns and children [2], [3], [4]. By contrast, in the wealthiest nations, infectious diseases are responsible for less than 5% of deaths [5]. In resource-poor countries where electricity, financial support, and skilled workforces are inadequate, the increasingly sophisticated medical technologies that allow rapid diagnosis of diseases are not widely available. The scarcity of government resources in combination with poor infrastructure hampers health care delivery, causing diagnosis to take days or even months [6]. Consequently, effective treatment is delayed resulting in an increase in both the mortality rate and economic burden on society.

There is growing interest in the development of appropriate, easy-to-adapt diagnostic technologies that can rapidly and accurately identify pathogens [7], [8], [9] [10]. Microfluidics-enabled testing is an option with the potential to improve global health and the status of epidemic control. It can offer several advantages [11]: 1) lower cost, 2) energy efficiency, 3) capacity to perform complex functions in a single device, 4) high sensitivity with small sample volumes, 5) lightweight and portability for in-field testing, and 6) relatively fast output. These unique characteristics make microfluidics a natural fit for portable point-of-care (POC) diagnostic systems [12], [13], [14], [15]. However, the application of microfluidics to medical diagnostic tools in developing countries is still evolving. A common approach for making diagnostic technologies a feasible option for the developing world is to make them completely self-contained and/or purely disposable (e.g., immunochromatographic strip) [15]. In addition to being simple and reliable, instruments must be robust enough so that little maintenance is needed, and operation can occur at a wide range (10 to 40°C) of ambient temperatures [15], [16]. Also, the device should be operational within the infrastructure of a resource-limited environment. Some diagnostic technologies have been developed and applied in the developing world, these include lateral flow tests for the diagnosis of diphtheria toxin and a number of sexually transmitted infections, including HIV [17], [18], [19], [20]. However, due to several limitations, lateral flow assays can not provide sufficient sensitivity and specificity required for accurate diagnostics in many cases [15].

There is an emerging literature describing a new class of devices that perform complicated biological manipulations with minimal instrumentation or instrumentation that requires limited or no laboratory support [21], [22], [23]. Several simple devices have been published that can be potentially applied in the developing world. For example, LaBarre and his colleagues [24] validated a first complete, non-instrumented nucleic acid amplification test (NAAT) by using a CaO heat source for a loop-mediated isothermal amplification (LAMP) assay. Also Wong *et al.* modified a hand-powered egg beater into a centrifuge to isolate human blood plasma from whole blood [6]. Several paper-based microfluidic devices have been reported for use in bioanalysis (e.g., level of glucose and proteins, pH, alkaline phosphatase) [25], [26], [27], [28].

One of the keys to preventing the spread of infectious diseases is to minimize the assay time and reduce lag times in patient treatment. A study by a panel of scientific experts in a variety low resource settings and diseases found that rapid, affordable molecular tests would be the most promising technology developed by 2012 [29]. Nucleic acid-based assays have been successfully implemented in recent years in many fields exploiting their rapid and accurate analysis (e.g., medical diagnostics, forensics, environmental analysis, and biodefense) [30], [31], [32], [33], [34]. Standard, tube based real-time polymerase chain reactions (qPCR) can produce results within 30 min [35]. However, the need for precise temperature control (i.e., use of a thermocycler), skilled personnel, and very clean conditions makes it challenging to utilize qPCR in many resource limited settings. Although a number of attempts have been made to simplify and integrate such PCR-based assays, methods published thus far generally require a thermocycler or an alternate precise heat controller capable of functioning at a wide range of temperatures. Hence, the development of a lower temperature, isothermal amplification assay is an attractive option.

**Figure 1:**
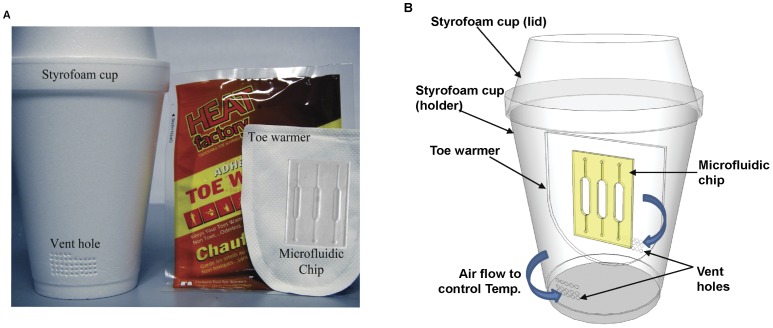
Components of the disposable DNA amplification platform. (A) Polymer-based microfluidic chip with multiple reaction chambers, a toe warmer, and Styrofoam cups; (B) Vent holes on the sides of the Styrofoam cup control the air supply to initiate and maintain the oxidation reaction in the toe warmer; the number of holes controls the temperature of the reaction chamber.

Here we describe a disposable DNA amplification platform based on isothermal helicase dependent amplification (HDA). This platform is capable of assaying DNA extracted from human stool specimens infected with *C. difficile*. The extracted DNA was obtained using a nucleic acid extraction protocol designed in our lab (unpublished data). As shown in **Figure 1**, the system consists of a low-cost thermoplastic microfluidic chip with three reaction chambers, a pair of toe warmers as heaters, and a Styrofoam passive temperature control system. The platform for DNA amplification consists of readily available inexpensive components and does not require a battery or electricity. Our design goal was to make the device user friendly so that people with minimal training are able to use it. In combination with emerging readout technologies [26], [36], [37], the system has the potential to make a great impact on disease diagnostic methodologies in resource-poor settings.

## Experimental Setup

### Design of the Microfluidic Chip

The microfluidic chip was constructed from six layers of cyclic olefin polymer (COP) film (Zeon Chemicals, Louisville, KY). The microfluidic channels were prepared by cutting 188 µm thick COP films using a Graft ROBO Pro CE5000-40 cutter plotter (Graphtec America Inc., Irvine, CA) [38]. The two sheets with inlet and outlet holes and the four sheets of cut film were stacked and thermally bonded in a hot press (Heated Press 4386, Carver, Wabash, IN) at 125°C for 20 min with 1.5 tons of pressure, followed by 10 min of curing at 137°C without any pressure. The inlet and outlet bridge channels are 188 µm high, 200 µm wide, and 10 mm long. The reaction chamber (0.75 × 2.5 × 15 mm) is able to hold an HDA reaction with a volume of 25 µl.

### Toe Warmer and Styrofoam Cup as a Heater

A toe warmer (Heat Factory, Vista, CA) was used as a heat source for the HDA reaction. It consists of a polypropylene bag containing iron, salt, activated carbon, cellulose, and vermiculite. When exposed to oxygen in the air, the iron powder oxidizes in an exothermic reaction. The salt serves as a catalyst while the carbon serves to disperse the heat. Vermiculite is added as an insulator to maintain the generated heat, and the cellulose is filler. Since the chemical components inside the commercially available toe warmer are fixed, the duration of the reaction (as well as the degree of heat generated) will mainly depend on air exposure and humidity supply. Relative humidity (RH) is important, since water on the surface of the iron particles can enhance the iron oxidation, and therefore, generate more heat. When the environmental humidity is high, more water vapour is available to promote oxidation. The amount of vapor that interacts with iron particles is dependent on the numbers of holes on the Styrofoam cup. The more holes, the more vapor particles can be trapped to react with iron. Therefore, by controlling numbers of holes on the Styrofoam cups, the temperature and duration of the reaction can be manipulated.

In our experiment, temperature control during a given experiment was governed by the number of 1 mm diameter vent holes, punched on opposite sides of a Styrofoam cup. To initiate the HDA reaction process, first two toe warmers were activated by the removal of their protective films. Next, the microfluidic reaction chamber, preloaded with the reaction mixture, was sandwiched between the warmers, and the entire assembly was placed inside the cup. Lastly, the Styrofoam cup was capped with another cup to complete the thermal chamber. The intra-cup temperature was monitored using a thermocouple attached to the microfluidic reaction chamber.

It is important to note that similar, and potentially more consistent results might be achieved using phase change materials [24], [39] rather than exothermic heat packs. Our choice of heat source was made solely based on convenience. This heat source worked well in our setting and can be easily adapted to others as described in the supplementary information.

### Control DNA Extractions and “Gold Standard” Diagnosis of *C. difficile*

To demonstrate the ability of our protocol to isolate and amplify DNA from patient samples without wall power or battery support, we chose an infectious diarrhea disease model. *C. difficile*-associated diarrhea (CDAD) is the most common nosocomial infectious diarrhea associated with prior antibiotic therapy. Two *C. difficile* toxins, toxin A (*tcdA*) and toxin B (*tcdB*), have a cytopathic effect on cell culture, and act synergistically to cause CDAD [40]. In this work, a tissue culture cytotoxicity assay (Toxi-Titre™ Plate-HF Cells, Trinity Biotech, Jamestown, NY) was used as the gold standard assay to pre-determine the existence of *C. difficile* infection in the stool samples. The discarded stool samples were obtained under an exempt institutional review board (IRB) protocol by the Microbiology Laboratory at Boston Medical Center and was stored at −80°C prior to gold standard testing and/or DNA extraction.

As a control DNA extraction process, we used a commercially available extraction kit. A QIAamp DNA stool mini kit (Qiagen) was used to extract stool genomic DNA following the manufacturer’s protocol. Briefly, 180 to 220 mg of stool was processed including a 95°C lysis step, and eluted in a final volume of 200 µl. The DNA concentration of the samples, as measured by NanoDrop 2000 c spectrophotometer (Thermo Fisher, Wilmington, DE), varied from 20 to 230 ng/µl, with the average concentration being 50 ng/µl. In this paper, 5 *C. difficile* positive and 3 negative (as determined by both the gold standard cytotoxicity test for *tcdA* and *tcdB*, and a tube based quantitative PCR for *tcdB*) were tested in chip to demonstrate the efficiency of our disposable DNA amplification platform.

### Extraction of Patient Stool Samples using our Portable System for Nucleic Acid Preparation (SNAP)

**Figure 2:**
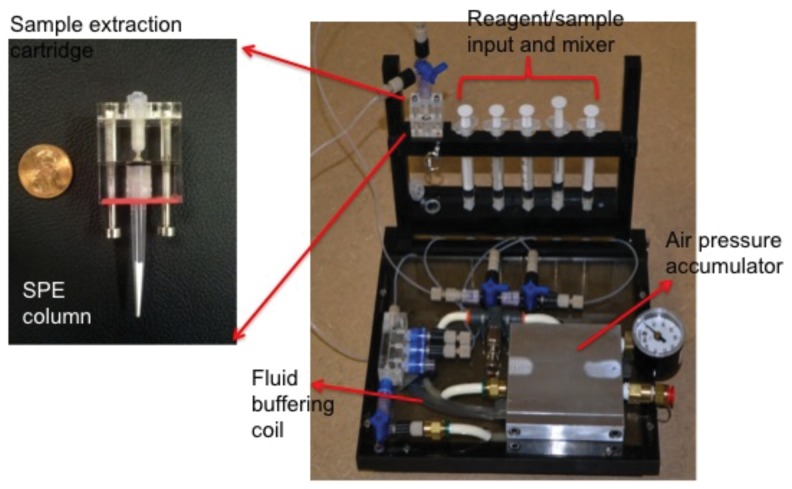
Picture of SNAP, the portable system for nucleic acid preparation.

In order to demonstrate the concept of minimally instrumented nucleic acid extraction, we also utilized a portable version of the microfluidic nucleic acid extraction instrument and protocols developed previously in our laboratory [41], [42], [43], [44]. The portable version of this extraction system is called SNAP-System for Nucleic Acid Preparation. It is an air pressure driven instrument powered by a standard bicycle pump. Stool samples (180–220 mg) were premixed with 700 µl 5.5 M GuSCN (Sigma-Aldrich, St. Louis, MO) lysis buffer for 30 min, spun at 14,000 rpm for 1 min. The supernatant was collected and introduced into the porous polymer monolith (PPM) column for selective nucleic acid capture. The PPM columns (**Figure 2**) were housed in unaltered FinnTip Universal 250 µl filter-less pipette tips (Thermo Fisher Scientific, Waltham, MA). These PPM columns were fabricated as previously described [41], [45] with some modification. Briefly, the pre-polymer monomer solution was prepared by mixing 125 µl Ethylene dimethacrylate (98%, EDMA), 125 µl Butyl methacrylate (99%, BUMA) (both Sigma-Aldrich, St. Louis, MO), 750 µl 1-dodecanol, and 8.0 mg azobisisobutyronitrile (AIBN). 25 µl of this solution was pipetted into each pipette tip. The pipette tips were incubated at 60°C for overnight, followed by washing with 200 µl of methanol and 150 µl of 95% ethanol to remove excess unreacted material. Finally, the solid phase extraction columns (SPEs) were dried in a fixture under air pressure for two minutes at 20±1.5% psi.

The SNAP (**Figure 2**) utilizes SPE to extract DNA from patient stool samples in a self-contained fluidic system. The SNAP device is a sealed fluidic system, which consists of four subsystems: a reagent/sample input and mixer, a fluid buffering coil, an air pressure accumulator, and a sample extraction cartridge. The DNA extraction protocol run on SNAP is essentially a modified Boom method [46] using the polymer monolith as the solid phase instead of a silica resin. The reagent/sample input is composed of five input ports designed for different buffers according to the sample preparation protocol. In these experiments, only one port is used, since only lysate and wash buffer (70% ethanol) were required. Extractions were run with 20±1.5% psi applied pressure, maintained by an in-line regulator. This in-line regulator steps the on-board pressure reservoir down to a constant 20±1.5% psi to maintain a constant pressure throughout the course of the extraction. Regulator operating pressure is set through the use of an external pressure gauge, and locked in place after calibration. DNA from the stool samples was eluted in three fractions of 40 µl nuclease free water. The extracted DNA was then amplified using our disposable thermoplastic chip and a heating system. The amplicons were examined via standard polyacrylamide gel electrophoresis (PAGE).

### Human GAPDH DNA Controls

To make sure that the extractions were successful and contained DNA, PCR control reactions were performed using primers designed to amplify the human GAPDH gene. These reactions were expected to be positive even in patient samples that were not infected with *C. difficile*, since all of the stool samples should contain human DNA. Each 25 µl reaction mixture consisted of 12.5 µl SYBR® Green PCR Master mix (Applied Biosystems, catalog #4309155), 0.1 µM GAPDH forward and reverse primers (Applied Biosystems, catalog #402869), 7 µl nuclease free water, and 5 µl DNA template. The thermal cycling conditions were as follows: 1 cycle of 95°C for 10 min, followed by 40 cycles of 30 s at 95°C, and 1 min at 60°C.

### HDA Amplification of *C. difficile*

In order to determine if the patient samples that tested positive by the gold standard cytotoxicity test would also test positive for *C. difficile* by HDA, an HDA reaction was set up using an IsoAmp III Universal tHDA kit from BioHelix (Beverly, MA). *C. difficile* genomic DNA from strain 630 (ATCC®, Manassas, VA) (0.5 ng/µl) was used as a positive control. The genomic DNA isolated from known negative patient stool samples was used as a check against false positives.

Primers for HDA toxinA (*tcdA*) were designed using PrimerQuestSM (Integrated DNA Technologies, Coralville, IA). The specificity of the primers for the *tcdA* gene was verified using NCBI BLAST (Accession #NC_009089.1) to assess their potential homology to other human and/or microbial genes. The primers used to amplify a 110 bp product were: *C. difficile tcdA* forward primer (SpeI) 5′-A CTA TAC TAG TGA TGT TGA TAT GCT TCC AGG TAT TCA C-3′ and reverse primer (AatII) 5′-GAA TGA CGT CTA TCA TTT CCC AAC GGT CTA GTC CAA T-3′. The primers contain SpeI and AatII restriction enzyme sites to allow the generated HDA amplicons to be gel isolated, cloned and sequenced to verify their identity. All the primers were synthesized by Integrated DNA Technologies. HDA conditions and reagent concentrations were optimized to obtain the final parameters described herein. In brief, 2.5 µl of DNA template was added to a 25 µl reaction mix containing 1X annealing buffer II, 40 mM NaCl, 4 mM MgSO_4_, 1.75 mM of IsoAmp® dNTP, 0.2 µM *C. difficile tcdA* forward primer, 0.1 µM reverse primer, and 12.75 µl nuclease free water.

### Limit of Detection of On-chip and In-tube HDA

To determine the limit of detection for *C. difficile* genomic DNA using our HDA assay, we spiked known concentrations of *C. difficile* genomic DNA into DNA extracts from *C. difficile* negative stool and performed HDA reactions. The negative stool sample extract material was included to ensure that the simulated samples contained the same (or similar) inhibitory molecules expected to be present in patient samples. Each 25 µl reaction contained 5 µl of DNA. Half of that (2.5 µl) was human specific DNA extract from negative (both *tcdA* and *tcdB* negative by cytotoxicity assay) stool extracted samples with an average concentration of 50 ng/µl. The other half contained titrations of ATCC genomic *C. difficile* specific DNA at known stock concentrations (effective masses ranging from 125 pg to 2.5×10^−3^ pg). The master mix was then split to perform both on-chip and in-tube HDA reactions to determine the detection limit of each methods. The in-tube HDA reactions were performed in triplicate using an ABI 2720 Thermocycler (Applied Biosystems, Foster City, CA) at 65°C for 30 min. The on-chip HDA reaction was performed using our toe warmer-Styrofoam cup disposable temperature controller at 65°C±2°C for 30 min. Total incubation time is average of 45 min, which includes the temperature ramps up to reach 65°C and 30 min incubation time. The microfluidic chip consisted of 3 channels, two of which contained the same concentration of *C. difficile* genomic DNA, while the third contained no template and served as a negative control. All experiments were repeated 2–3 times to confirm the results.

### Polyacrylamide Gel Analysis

PCR products generated from amplification of the human GAPDH gene (226 bp) and HDA *tcdA* amplicons (110 bp) were analyzed on a 12% non-denaturing polyacrylamide gel using MspI digested pBR322 plasmid (New England Biolabs, Beverley, MA) as a size marker. Gels were run at 75 V for 135 min then incubated in 1X TBE buffer with 0.001X SYBR Green (Invitrogen, Carlsbad, CA) at room temperature for 30 min. Finally gel photos were taken under UV light using Molecular Imager PharosFXTM System (Bio-Rad, Hercules, CA).

### Validation of HDA toxinA Amplicons

To verify the identity of the HDA *tcdA* product, the 110 bp amplicon was cloned and sequenced. Briefly, HDA products were subjected to phenol chloroform extraction followed by ethanol precipitation and then digested with restriction enzymes SpeI and AatII. Digested fragments were purified using PAGE and ligated to the pGEM®-T Easy vector (Promega). Ligation was transformed into Top10 chemically competent *E.coli* (Invitrogen). DNA plasmids were isolated using Qiagen QIAprep spin mimiprep kit (Qiagen). Two to four clones were selected for analysis and subsequent sequencing with M13 reverse primer (sequence primer) by Genewiz DNA Sequencing Center (Boston, MA).

## Results

### Temperature Stability of Disposable Heat Source

**Figure 3:**
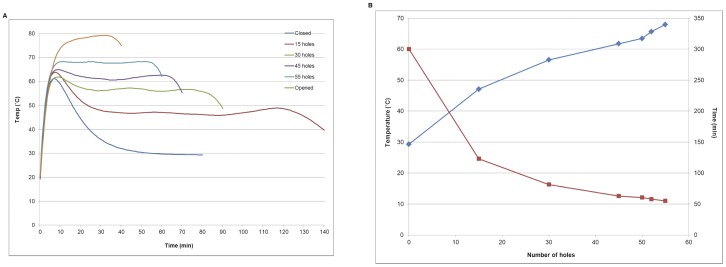
Temperature stability inside the Styrofoam cups with different numbers of holes punched on both sides. Temperature was measured on a microfluidic reaction chamber sandwiched between two toe warmers. The platform is able to maintain the temperature at 65°C±2°C for more than 55 min.

To examine the capability of our toe warmer-Styrofoam cup system to maintain a constant temperature, activated toe warmers were placed in the thermal chamber with vent holes (diameter of 1 mm made using a standard metal punch) on opposite sides of the chamber as shown in **Figure 1**, and temperature readings were recorded at thirty second intervals using a thermocouple attached to the microfluidic chip reaction chamber. We first explored the relationship between the number of vent holes (0, 15, 30, 45, 55 and fully opened with no vent holes or top cup) and the temperature stability of the reaction chamber. As shown in **Figure 3(A)** (external temperature was 20°C with ambient humidity 25±2.5%RH), the time it took the chamber temperature to reach steady state was inversely proportional to the number of vent holes. In the closed cup with no holes, the temperature reached steady state after 50 min, while only 22 min was required when the cup had 15 vent holes. A greater number of vent holes causes the oxidation reaction inside toe-warmer to proceed faster resulting in the generation of more heat per unit time. However, a faster oxidation reaction also leads to a more rapid consumption of the iron particles inside the toe warmer. Therefore, as shown in **Figure 3(B)**, we found the greater the number of vent holes resulted in a corresponding decrease in the length of time quasi steady state temperature was maintained. The open cup (without vent holes and top cup) never reached a steady state temperature and peaked near 80°C at approximately 30 min. In contrast, 55 vent holes resulted in a near steady state temperature of 65°C, the optimum temperature of HDA reaction that was maintained for greater than 55 min. These experiments were repeated at different ambient temperatures **(Figure S1)**.

It is important to qualify that these results are for our particular setting and ambient conditions. We explored the stability of the system further for different ambient conditions, and these results are presented in more detail in the supplementary information. Briefly, we looked at two additional external temperatures as well as different humidity environments. As shown in **Figures S2A and S2B**, the experiments above were run again in temperatures of 30°C and 40°C with the humidity of 25±2.5%RH. For these conditions, 46 and 20 vent holes, respectively were needed in the Styrofoam cup chamber to maintain a 65°C±2.5°C for more than 30 min. In order to study the effect of humidity on our device, the experiments were also run under temperatures of 30°C with the humidity of 65±1.5%RH and 80±0.8%RH. As shown in **Figures S3A and S3B**, 39 and 35 vent holes, respectively were needed in the Styrofoam cup chamber to maintain a 65°C±2.5°C, and the duration of this quasi steady state temperature is longer (more than 80 min) when compared with **Figure S2A** (30°C with the humidity of 25±2.5%RH). Similar trend (**Figures S4A and S4B**) was found when the external temperature was 40°C with the humidity of 58±0.8%RH and 82±0.4%RH, respectively. For these conditions, 10 and 7 vent holes, respectively were needed in the Styrofoam cup chamber to maintain a 65°C±2.5°C for more than 60 min. We also tested the effect of varying humidity (**Figures S5A and 5B**) when the external temperature is 20°C. Again, a similar trend was found, and 48 and 46 holes are required respectively to keep the temperature 65°C±2.5°C for HDA reaction. Overall, as humidity increases, fewer holes are required and the duration of steady state temperature is longer.

### Microfluidic Channel HDA

The manufacturer’s protocol recommended conditions for HDA in-tube reactions is 65°C for 90 min. We repeated HDA in-tube reactions using our optimized HDA amplification recipe for 30, 60, and 90 min. time points, and found that a 30 min. reaction was sufficient to reliably amplify the *C. difficile tcdA* product (**Figure S2**). We first tested our toe warmer-Styrofoam cup platform by amplifying purified ATCC *C. difficile* genomic DNA (0.5 ng/µl). HDA reaction products were subjected to polyacrylamide gel analysis and then cloned and sequenced as described above to confirm their identity. The sequencing results showed that these were true positive products. The ATCC *C. difficile* genomic DNA was used as a positive control for each on-chip HDA reaction.

**Figure 4:**
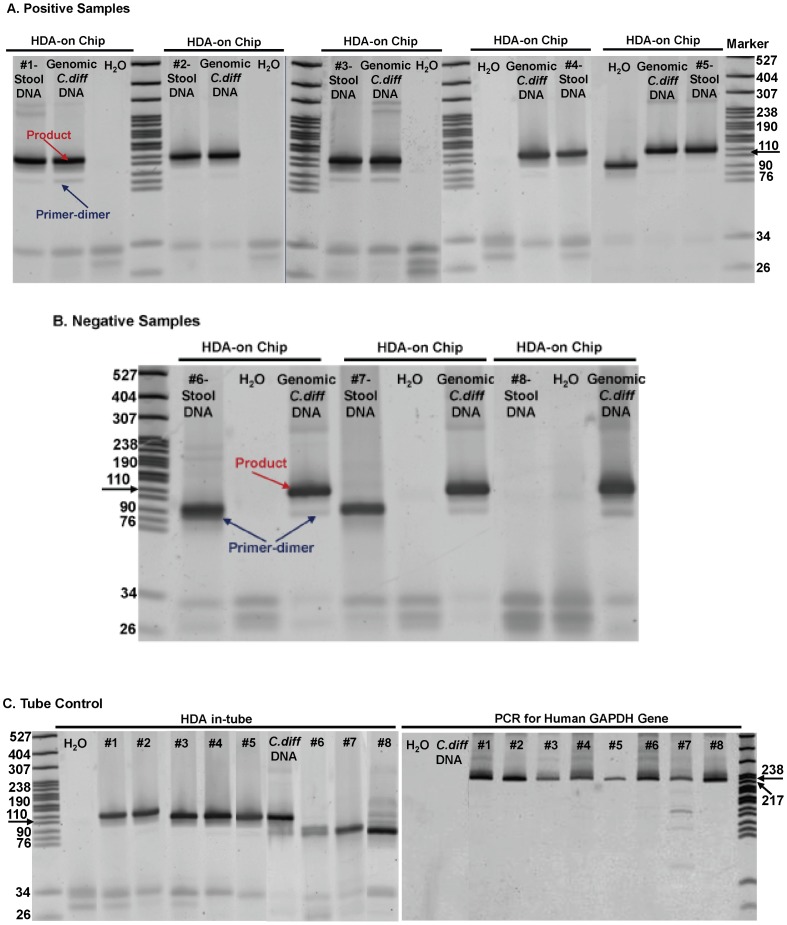
Gel electrophoresis analysis of the HDA on-chip amplicons using 12% polyacrylamide gel with MspI digested pBR322 as marker. (A) five positive human stool DNA samples infected with *C. difficile*; (B) 3 negative human stool samples; (C) HDA in-tube as a control, and PCR control reactions to quantify the amount of DNA in the stool extract using human GAPDH as a housekeeping gene.

The next step was to use the platform to amplify DNA extracted from human stool samples. The concern here is that the ratio of target DNA to total DNA extracted may be extremely low. In addition, inhibitors present in extracted stool samples may negatively affect the HDA assay, by decreasing its sensitivity and or specificity. Extracted stool DNAs from 8 patients (5 positive, 3 negative by the cytotoxicity assay and confirmed with qPCR) were amplified in the disposable platform. For each three channel chip, one channel was used for the test patient sample, one for the positive control (commercial *C. difficile* DNA (0.5 ng/µl)), and one for the negative control. As shown in **Figure 4(A)**, polyacrylamide gel analysis confirms that the toe warmer-Styrofoam cup platform was effective at amplifying the appropriate *tcdA* products from both stool DNA samples (#1, #2, #3, #4 and #5) and commercial genomic DNA, but no product was generated in the negative control reaction. In addition, as shown in **Figure 4(B)**, none of the 3 cytotoxicity negative stool samples (#6, #7 & #8) amplified a product. The HDA on-chip reaction results were consistent in each case with the corresponding reaction in-tube (**Figure 4(C)**) and cytotoxicity assay. To confirm the integrity of the genomic DNA in the *C. difficile* negative stool samples, PCR was performed to detect the human GAPDH gene. ATCC *C. difficile* genomic DNA and nuclease free water were used as templates for negative control reactions. The expected human GAPDH gene products (**Figure 4(C)**) were detected in both negative and positive stool samples. Reactions containing the ATCC *C. difficile* genomic DNA and nuclease free water were negative. This confirms that negative stool samples contained intact genomic DNA.

### SNAP Extracted DNA

**Figure 5:**
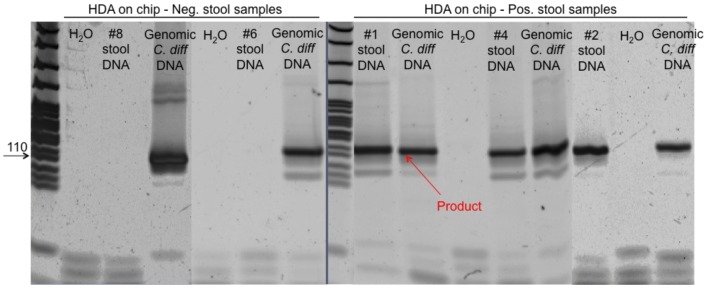
Gel electrophoresis analysis of the HDA on-chip amplicons that was extracted via SNAP.

The patient stool DNA extracted via SNAP was collected and amplified using our disposable thermoplastic chip and heating system. As shown in **Figure 5**, none of the negative stool samples (#6, #8) amplified a product, while the positive stool samples (#1, #2, #4) was effectively amplified. This result is consistent with the result of DNA extracted by Qiagen kit. And further demonstrate that the sample preparation and amplification can be done non-lab instrumented.

### Limit of Detection

**Figure 6:**
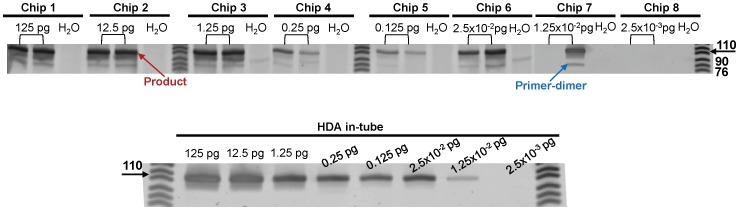
Sensitivity of HDA on-chip vs. in-tube reaction. HDA mixture with decreasing amounts (125 pg –2.5×10^−3^ pg) of *C. difficile* purified genomic DNA as the template. Then HDA was performed both on-chip (in the power free system) and in-tube (using a thermocycler). The product yields were compared using PAGE showing the correct size of 110 bp.

The performance of the on-chip and in-tube HDA reactions varied in terms of the lower limit of detection. From **Figure 6**, the on-chip reaction using the disposable platform produced a lower limit of detection just above 1.25×10^−2^ pg. As listed in Table 1, for each concentration of ATCC *C. difficile* genomic DNA, the HDA reaction was repeated at least 6 times. We found that at high *C. difficile* DNA concentrations (above 0.125 pg), the limit of detection for HDA on-chip was comparable to HDA in-tube reactions. However, as the amount of spiked *C. difficile* DNA decreased to 2.5×10^−2^ pg, the in-tube *vs.* on-chip results diverged (**Table 1**). In the tube reactions, below 2.5×10^−2^ pg, not all of the replicates resulted in amplified product. For the chip-based reactions, this lower limit was 1.25×10^−2^ pg. Neither the in-tube or on-chip HDA reaction could detect *C. difficile* genomic DNA below 10^−3^ pg/µl (amount in reaction is 2.5×10^−3^ pg). Thus, the disposable DNA amplification platform performed similarly to the standard tube reactions in this case. In terms of cost, the disposable platform is capable of providing enough heat, and stable heat for the reaction for a much lower cost (<$1 US) compared to the standard tube method where a commercial thermocycler is used as a heat source.

**Table 1:** Sensitivity of HDA on-chip reaction *vs.* in-tube reaction.

C. difficile in 25 µl		HDA in-tube		HDA on-chip	
Conc. (pg/µl)	Mass (pg)	No. reactions	No. positives	No. reactions	No. positives
50	125		6		6
5	12.5		6		6
0.5	1.25	6	6	6	6
0.1	0.25		6		6
5×10^−2^	0.125		6		6
1×10^−2^	2.5×10^−2^	6	4 (66%)	6	6 (100%)
5×10^−3^	1.25×10^−2^	6	2 (33%)	6	3 (50%)
10^−3^	2.5×10^−3^	6	0	6	0

## Discussion

This proof-of-concept study highlights a disposable thermoplastic chip and a heating system that combines a toe-warmer and Styrofoam to perform the isothermal HDA reaction aimed to detect the presence of *C. difficile* in DNA extracted from patient stool samples. Total incubation time is average of 45 min, which includes the temperature ramps up to reach 65°C and 30 min incubation time. In addition to the extraction of DNA by Qiagen kit, we also showed that a portable device (SNAP) developed in our lab is able to extract DNA from patient stools without the use of electrical power. The extracted DNA can be amplified by our disposable amplification system. The combination of both setups and low material cost make the protocol promising for further integration with a direct read-out system for eventual application in low resource settings.

The HDA reaction often exhibits non-specific amplification as the reaction reaches steady state, which can sometimes lead to a long nonspecific product that can be confused with target products [47]. To avoid this problem we optimized the reaction time to both amplify the correct product and limit the non-specific amplification. Via this process, we were able to significantly lower the background as is evident on the PAGE gels. Recently [48] it was shown that the HDA reaction time can be decreased to 22 min for the detection of *Mycobacterium tuberculosis* by optimizing the primer and the enzyme concentrations. In our case, 30 min was suitable to amplify DNA extracted from patient stool samples while limiting background signal. The on-chip reaction does generate a primer-dimer product of 76 to 90 bp (**Figure 6**). Unreacted primers (34, 26 bp) were also visible at an amplification time of 30 min. Primer-dimers were not seen as often when the genomic *C. difficile* DNA and extracted patient stool samples were amplified in-tube. This result suggests that the undesired primer-dimer formation may be related to the high surface to volume ratio environment in the chip. In both the in-tube and on-chip reactions, primer-dimers sometimes formed in the negative control reactions (no template). Adding DMSO, adjusting the amount of MgSO_4_, and treating COP channel surface with the blocking agents to prevent enzyme loss to provide lower detection limits and limit the formation of primer dimers, are other ways to optimize the amplification assay. In **Figure 6**, the detection limits of in-tube *vs.* on-chip results diverge when the concentration of *C. difficile* genomic DNA decreases. This might be a result of chip to chip variations during the fabrication process. The thermo cycler method also clearly has the advantage in terms of temperature control. Also variations in pipetting and sample collecting might also affect the observed reaction efficiencies at lower concentrations. Not all of the experiments were run on the same day, and several lot numbers of the HDA enzyme kit were used.

The existing toe warmer-Styrofoam cup setup provides a constant 65°C±2°C for 55 min, which is more than sufficient for the HDA amplification to occur within the context of a controlled lab (ambient) environment. All of the data in **Figure 2** was obtained under the same ambient conditions. In the field, ambient conditions can vary widely, and this needs to be taken into account (i.e. external temperature, humidity) when scaling up this technology. In our supplemental study, the external temperature (20–40°C) and humidity (25±2.5%RH − 80±2.5% RH) are varied. We found for the same external temperature, the higher the external humidity, the fewer holes are required to reach 65°C±2°C. Also the duration of quasi steady state temperature is longer. This result is likely due to the fact that when the humidity is high, there will be more water vapour available. If there are too many holes, oxidation will occur too quickly to consume the iron, thereby decreasing the duration of the steady state regime. Therefore, an optimum number of holes is important. On one hand, the holes need to provide enough humidity and O_2_ to promote the oxidation. On the other hand, the reaction can happen too quickly, overheating the enzyme and halting the reaction. For the same external humidity, the higher the external temperature, the fewer holes are required to reach 65°C±2°C for the HDA reaction. The higher the temperature, the faster the reaction. By decreasing the number of holes, less O_2_ is provided, and oxidation rate slows down. Our long-term goal is to provide a cross-reference chart so users can tailor the required airflow to maintain 65°C±2°C for 30 min at different locations ranging from 20–40°C. As mentioned earlier, there are other electricity and battery free methods for maintaining constant temperature in the field. Most notably, phase change materials have been used to achieve this goal successfully for a number of biochemical assays. Likely the phase change material approach will be more successful and reliable than the exothermic reaction method presented here for many settings, but both are viable for use in the field.

Though we have successfully used the SPE columns without centrifugation steps with urine, blood and nasowash, stool samples are a rather special case. They vary greatly in quality, and in order to obtain suitable analytical samples, often formed stools have to be used. The centrifuge step is used to prevent the solid stool particles from clogging the SPE channel. And of course, the centrifuge is powered. Although it is certainly possible that a battery or hand powered centrifuge may be suitable for removing enough particulate to inhibit clogging, we did not do that work here.

We did however, look at how the device would perform without any centrifugation step at all. In order to reach the goal of using no battery or wall power, we tried to eliminate the centrifuge step by replacing it with filtration. After adding lysis buffer, instead of using centrifugation to remove the solid particles, the lysate was filtered by using a filter with 0.2 µm pore size. The stool specimens may be liquid, soft, semiformed, and formed. For these samples, especially those formed or semiformed, it is hard to remove the solid particles by filtration alone. These samples clogged the column. However, for the samples which are more liquid phase, we were able to isolate DNA without using centrifuge pre-extraction step and did amplify that DNA using our toe warmer-Styrofoam setup (shown in **Figure S6**). This demonstrates our nucleic acids extraction procedure can be electricity free if the sample is liquid phase. Future efforts will focus on making this device completely electricity free, which might involve several filtration steps, optimizing pore size of SPE column to avoid the clogging issue or combining a portable centrifuge.

Although we looked at clinical samples here, it is difficult to clearly articulate the lower limit of detection (LLOD) for the clinical case. Little work has been done to assess the molecular (DNA) LLOD for *C. difficile*. The lack of LLOD for molecular data is due to the longstanding use of a protein biomarker for diagnosis (toxin culture assay). It has been assumed until recently that if one genome of *C. difficile* was detectable, then the person should be considered sick. More recently, as molecular tests have become available, this assumption is being called into question. It appears from some studies that a patient can be colonized with *C. diffcile*, but not sick. This idea is still controversial among clinicians. Since it is very difficult to grow the bug in culture, it is not possible to conduct LLOD tests with stool samples spiked with known amounts of bug. Thus, we used genomic DNA to achieve this task. We acknowledge that for this particular organism, there is a gap in the data in terms of defining a clinically relevant LLOD.

In summary, genomic DNA extracted from patient stool samples was successfully amplified in the absence of a thermocycler. The previous work [41], [42], [47] from our group has demonstrated on-chip extraction of complex biological sample (e.g. blood, stool) using integrated micro solid phase extraction (µSPE) columns. Our next challenge will be to design an integrated cartridge with the capacity to perform DNA extraction from a stool sample, nucleic acid amplification, and then directly display the results by simple visual inspection. Since the HDA reaction only requires one temperature of 65°C, (in contrast to PCR, where a 95°C step is required), the relatively expensive COP film that has glass transition temperature of 136°C can be replaced with a less expensive material like poly(methyl) methacrylate. A low cost readout using visual inspection has been developed by BioHelix, and relies on a cassette-based, lateral flow approach to identify *C. difficile* [26]. In their method, a lateral flow strip is pre-coated with two types of antibodies that react with two specific labelled probes in HDA amplicons respectively to serve as control (C) line and test (T) line. The amplicon is negative when only C line is visible, and it is positive when only T line or both the T and C line are visible. Combining our on chip extraction technology with a visual readout like the BioHelix platform would yield a very low cost and robust integrated diagnostic.

### Conclusions

We developed a platform composed of a microfluidic chip, toe warmers and Styrofoam cups to perform isothermal DNA amplification without electrical power. By punching various numbers of holes on cup, the amount of supplied air was controlled so that the desired temperature could be maintained. The capability of the system to maintain a constant temperature was investigated. While more holes shortened the time to reach the steady temperature, the duration of the stable temperature was decreased. The temperature measurement indicated that a system with 55 holes can hold 65°C±2°C for more than 55 min. By optimizing the concentration of enzyme and primers, the amount of time needed for the specific HDA reaction used here as a proof of concept can be decreased to 30 min, while producing less non-specific amplicons.

We performed isothermal amplification of DNA extracted from patient stool samples, and specific identification of *C. difficile* was achieved based on HDA for *tcdA*. The results were consistent with the gold standard cytotoxicity assays for *C. difficile* in stool. We also demonstrated that our disposable platform functions comparably to a standard method using a thermocycler as a temperature control with similar detection limits for *C. difficile* DNA in patient samples. This work is a step toward the fabrication of an inexpensive, handheld, point-of-service disposable diagnostic assay. Continuing work includes integration of on-chip stool extraction and readout to make a completely handheld device.

## Supporting Information

Figure S1 **Temperature stability inside the Styrofoam cups with different numbers of holes under A) 30°C and B) 40°C.**
(TIF)

Figure S2 **HDA in-tube reactions for different total amplification times: 30, 60, 90 min.** Note that primer-dimers started to form when the reaction is longer than 60 min.
(TIF)

Figure S3 **Temperature stability inside the Styrofoam cups with different numbers of holes at 30°C under A) RH 65±1.5% and B) 80±0.8%.**
(TIF)

Figure S4 **Temperature stability inside the Styrofoam cups with different numbers of holes at 40°C under A) RH 58±0.8% and B) 82±0.4%.**
(TIF)

Figure S5 **Temperature stability inside the Styrofoam cups with different numbers of holes at 20°C under A) RH 40±0.9% and B) 72±0.6%.**
(TIF)

Figure S6 **PAGE gel showing SNAP extracted DNA amplified using the Styrofoam heater without a pre-centrifugation step.** Note that this experiment was only successful for liquid phased stool. Formed and semiformed samples clogged the SPE columns before extraction was complete.
(TIF)
