# Supplementary figures and images for: Low Cost Extraction and Isothermal Amplification of DNA for Infectious Diarrhea Diagnosis
Source: PLoS One. 2013 Mar 28;8(3):e60059. doi: 10.1371/journal.pone.0060059 (PMC3610934; doi:10.1371/journal.pone.0060059)

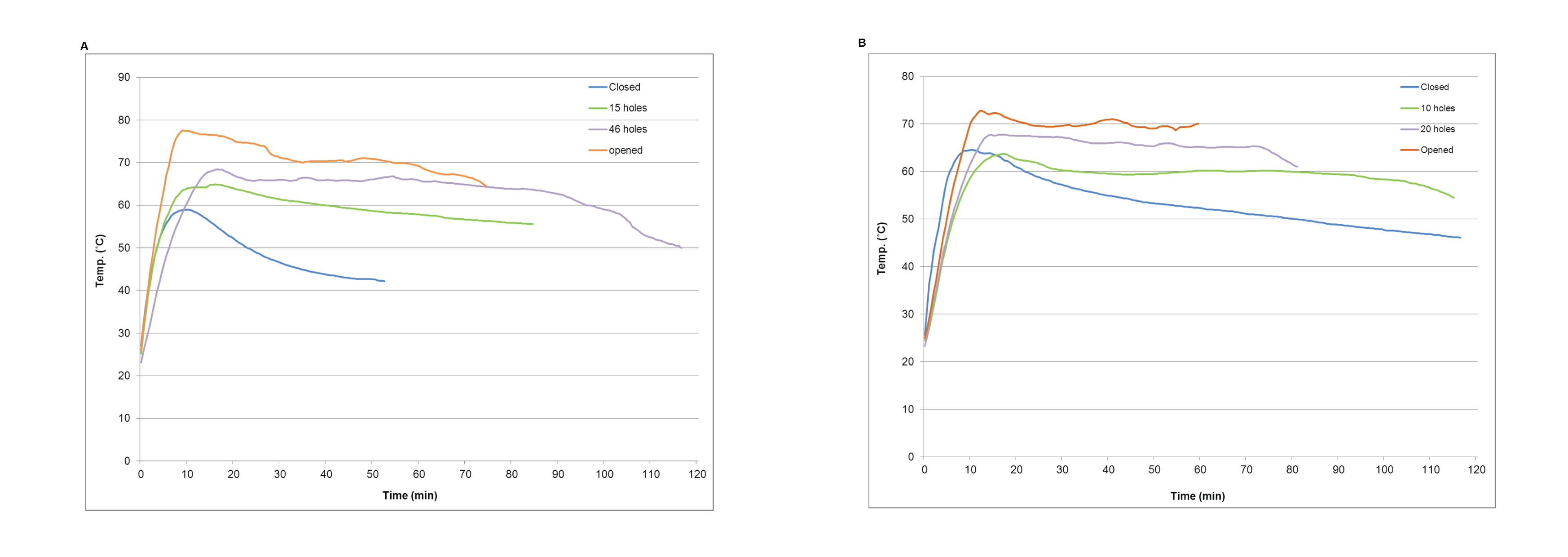

Supplement: Figure S1 — Temperature stability inside the Styrofoam cups with different numbers of holes under A) 30°C and B) 40°C. (TIF) [file pone.0060059.s001.tif]

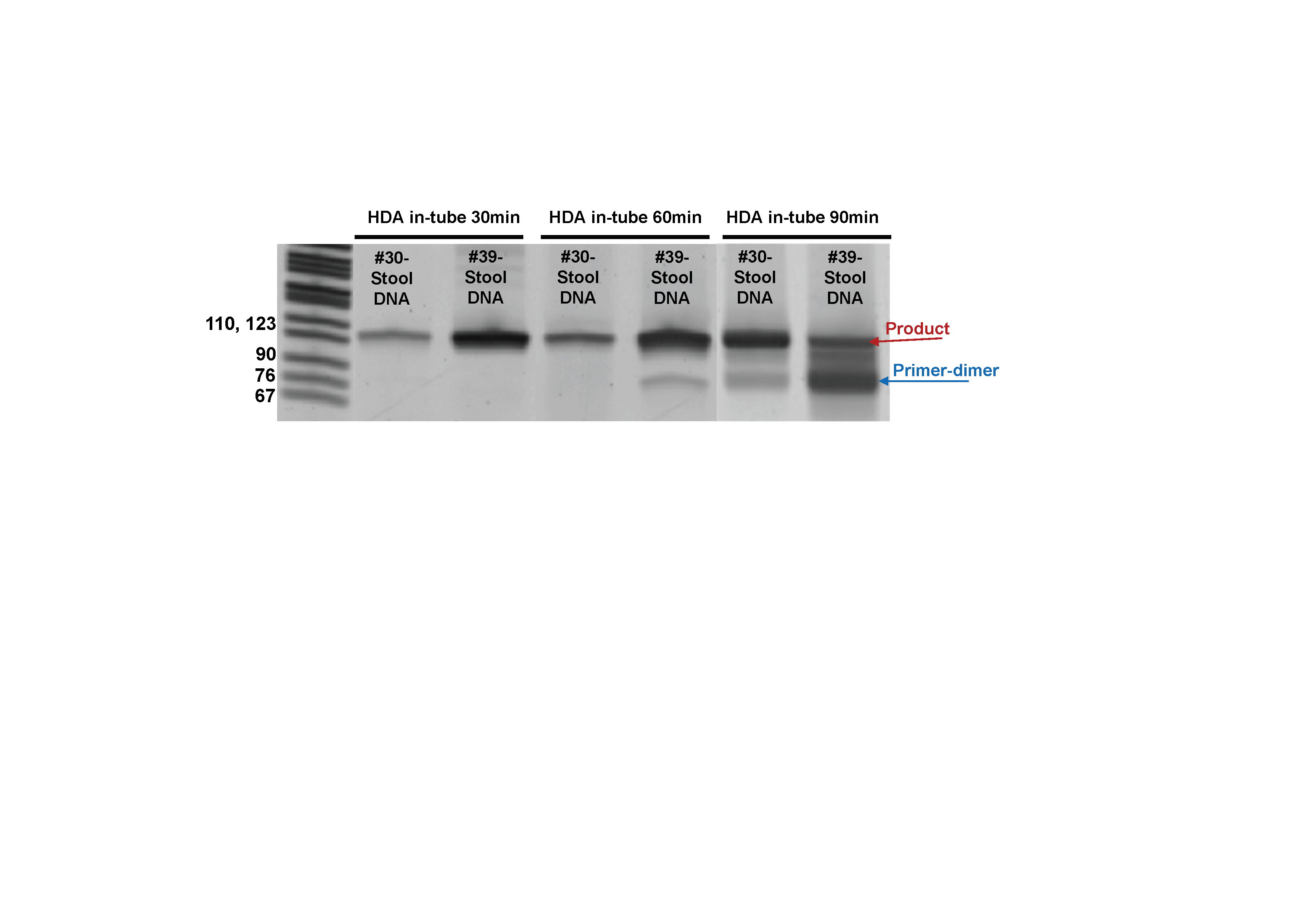

Supplement: Figure S2 — HDA in-tube reactions for different total amplification times: 30, 60, 90 min. Note that primer-dimers started to form when the reaction is longer than 60 min. (TIF) [file pone.0060059.s002.tif]

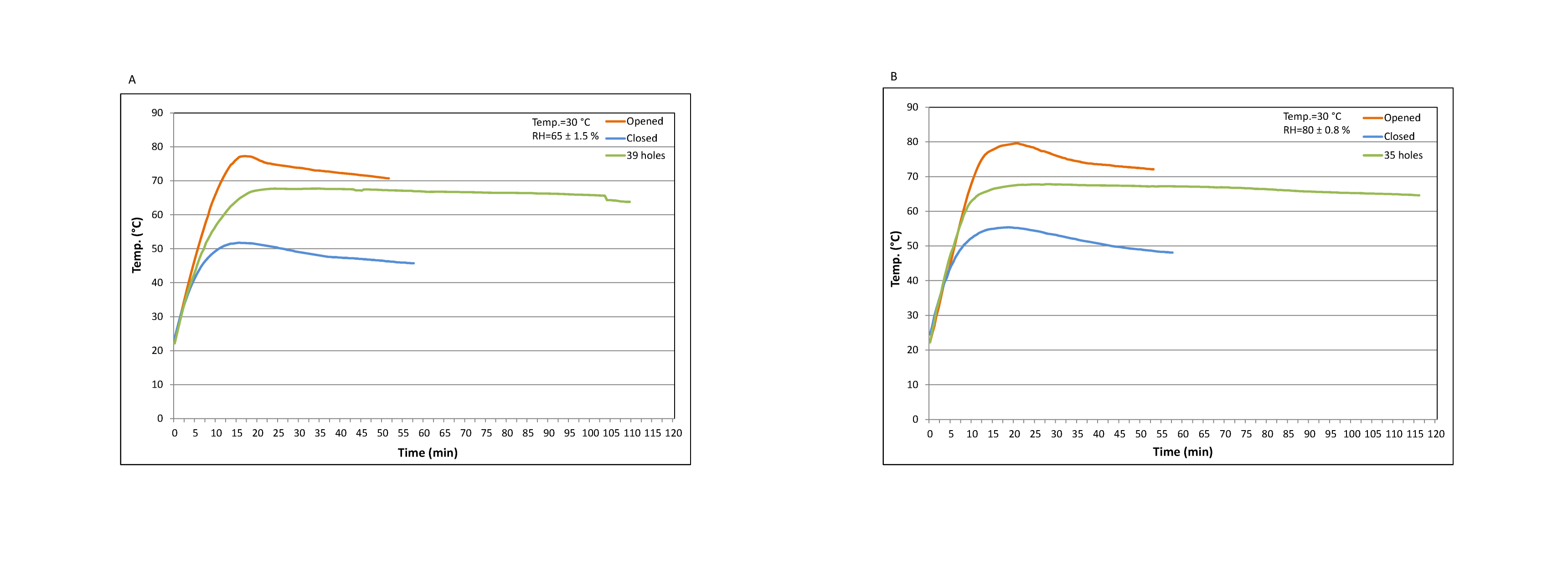

Supplement: Figure S3 — Temperature stability inside the Styrofoam cups with different numbers of holes at 30°C under A) RH 65±1.5% and B) 80±0.8%. (TIF) [file pone.0060059.s003.tif]

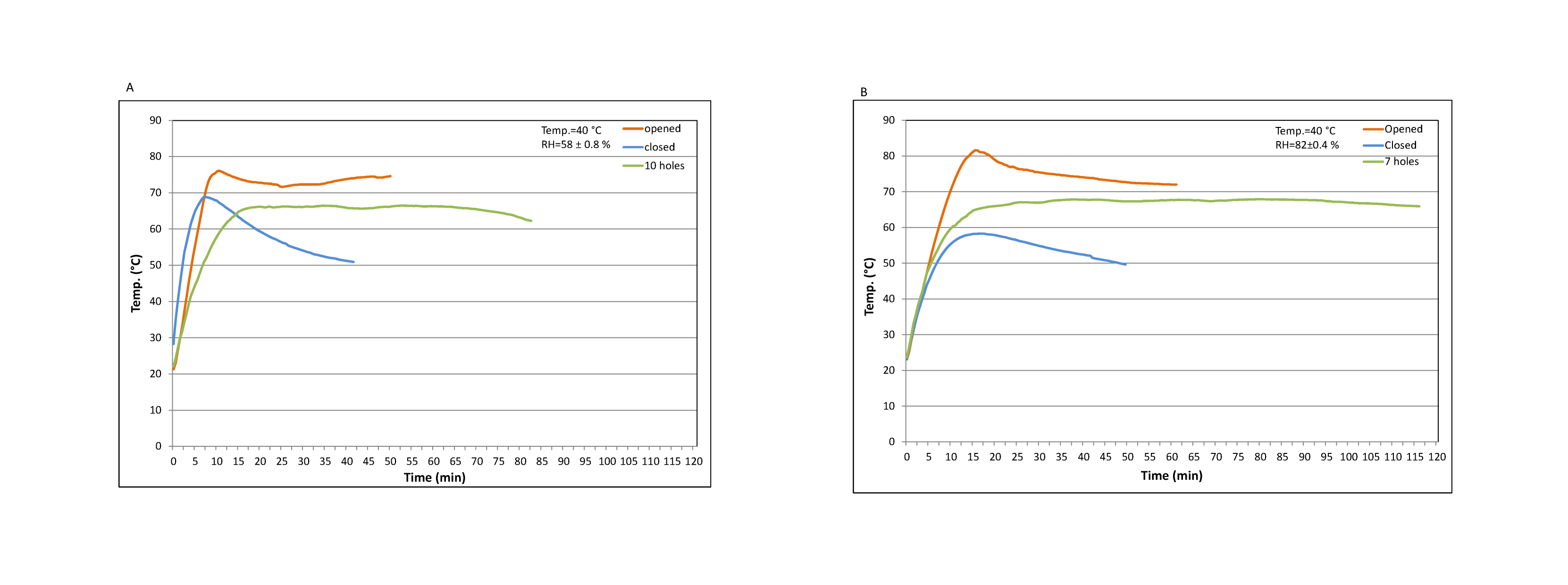

Supplement: Figure S4 — Temperature stability inside the Styrofoam cups with different numbers of holes at 40°C under A) RH 58±0.8% and B) 82±0.4%. (TIF) [file pone.0060059.s004.tif]

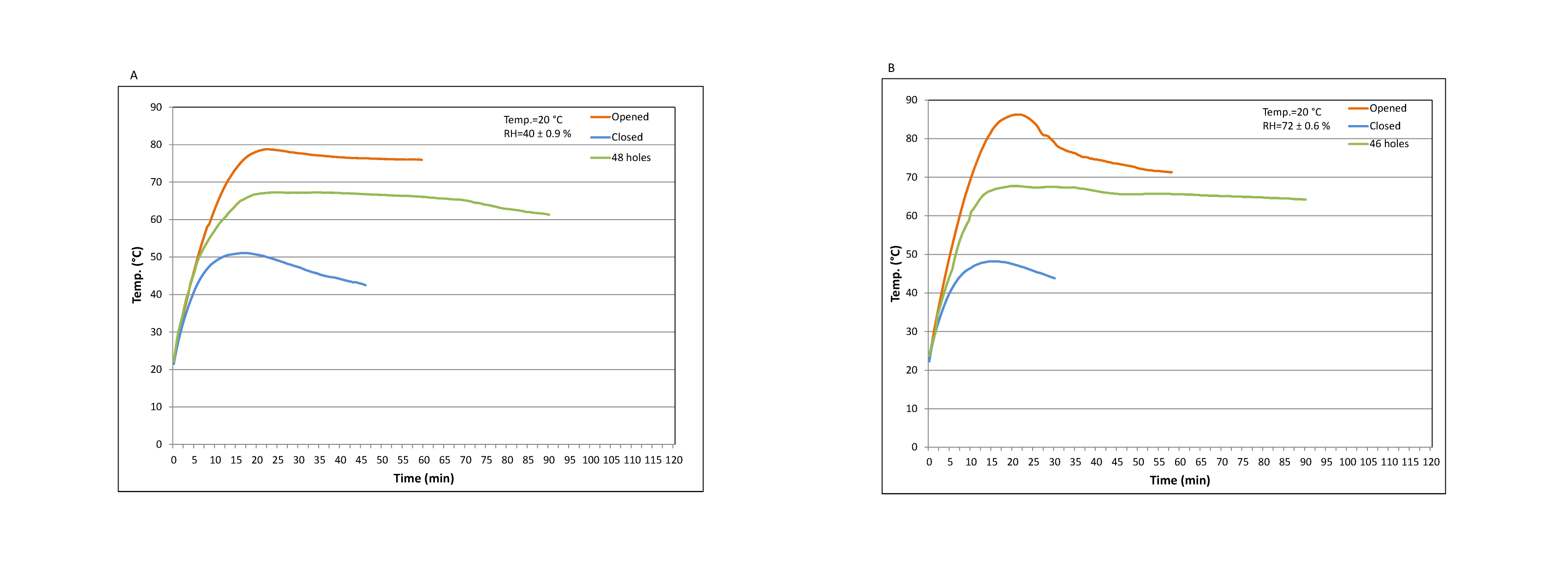

Supplement: Figure S5 — Temperature stability inside the Styrofoam cups with different numbers of holes at 20°C under A) RH 40±0.9% and B) 72±0.6%. (TIF) [file pone.0060059.s005.tif]

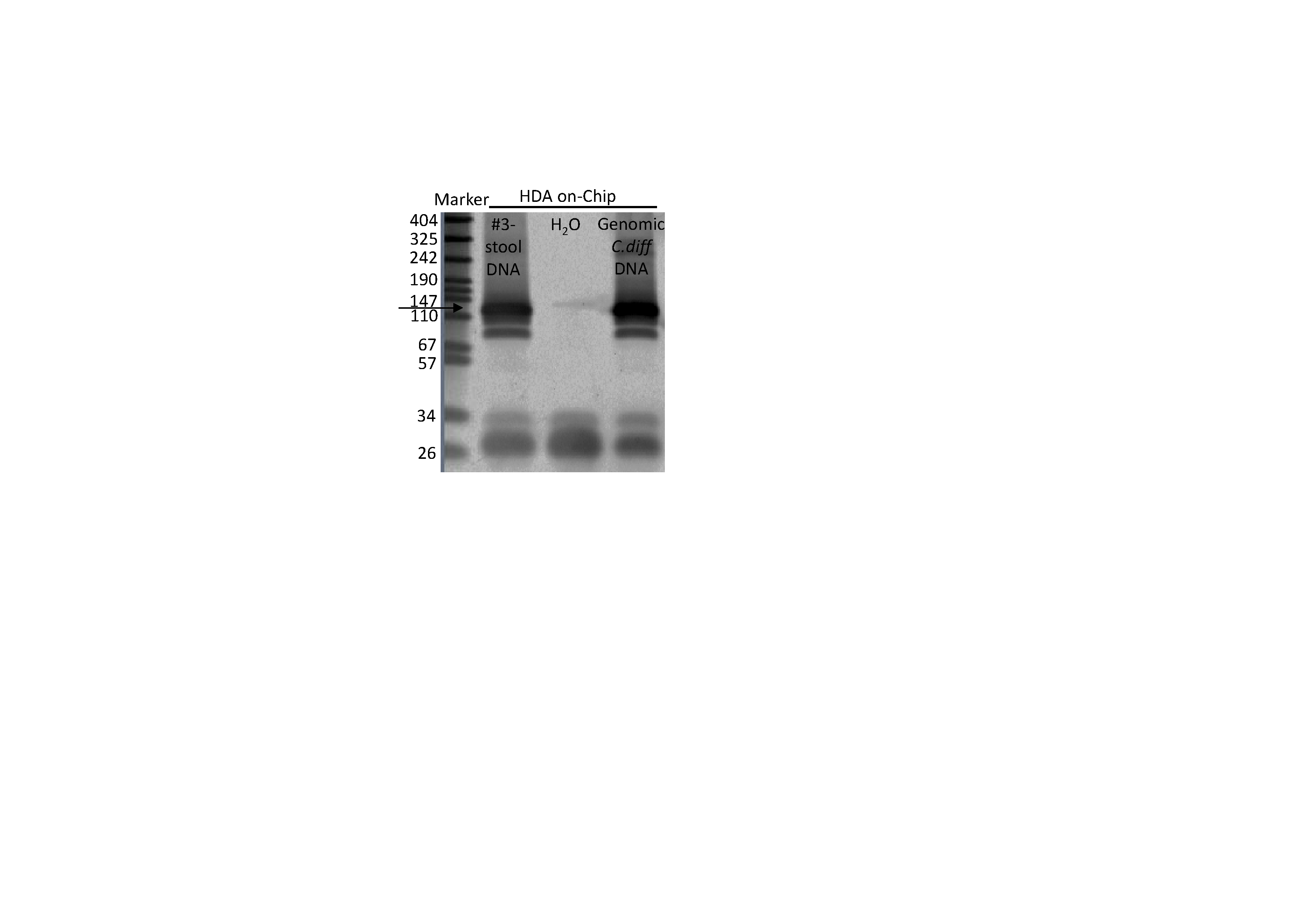

Supplement: Figure S6 — PAGE gel showing SNAP extracted DNA amplified using the Styrofoam heater without a pre-centrifugation step. Note that this experiment was only successful for liquid phased stool. Formed and semiformed samples clogged the SPE columns before extraction was complete. (TIF) [file pone.0060059.s006.tif]
